# Four odorants for olfactory training are enough: a pilot study

**DOI:** 10.1007/s00405-024-08930-4

**Published:** 2024-09-06

**Authors:** Nicole Power Guerra, Emely Kruschwitz, Dietmar Krautwurst, Thomas Hummel

**Affiliations:** 1 Department of Otorhinolaryngology, Faculty of Medicine Carl Gustav Carus, Smell & Taste Clinic, Technische Universität Dresden, 01307 Dresden, Germany; 2 Leibniz-Institute for Food Systems Biology at the Technical University of Munich, 85354 Freising, Germany

**Keywords:** Olfactory training, Seven odors olfactory training, MOCA test, Olfactory dysfunction, COVID-19 related smell loss

## Abstract

Background: Olfactory training (OT) is commonly used for the treatment of olfactory disorders. Nevertheless, there is an ongoing debate about the most effective OT regimen. We aimed to compare the effects of OT with 7 items (rose, lemon, eucalyptus, cloves, stewed apple, balm, mint) to 4-item-OT (rose, lemon, eucalyptus, cloves) over 3 months. Methods: Participants were 40 patients with olfactory dysfunction receiving 4-item-OT or 7-item-OT and 60 gender- and age-matched individuals with normal sense of smell receiving no OT, 4-item-OT, or 7-item-OT. Before and after the OT we assessed n-butanol odor thresholds, discrimination, and identification (TDI score), additionalthresholds for (R)-(-)-carvone, β-damascenone, salicyclic acid benzylester, the degree of phantosmia and parosmia, cognitive function, and ratings of olfactory function. Results: In both patient groups, the TDI score increased with the use of OT, regardless of the number of odors used (*p* < 0.001; 3.48 ± 4.21 and lower than control groups). The clinically significant increase of 5.5 points in TDI score correlated with change of ratings of parosmia (*r* 0.62; *p* < 0.01) and with ratings of olfactory dysfunction (*r* = 0.51; *p* < 0.05). Conclusion: Concluding, OT over a 3-months period with 4 or 7 odors appears to produce similar results, although the sample size has to be considered.

**Supplementary Information:**

The online version contains supplementary material available at 10.1007/s00405-024-08930-4.

## Introduction

The human sense of smell received unprecedented social and scientific attention once smell loss appeared to be a significant symptom of COVID-19 [1]. Considering the negative consequences of chemosensory dysfunction for the quality of life [2], an effective treatment for olfactory dysfunction (OD) is needed [4]. In order to assess the impact of treatment, quantification of olfactory function can be assessed by measuring patient’s odor threshold (T), discrimination (D), and identification (I; TDI score) [3]. Olfactory training (OT) has been shown to be effective not only in the treatment of OD associated with COVID-19 [4], but also for post-traumatic and idiopathic OD [5, 6]. The effectiveness of OT ranges from 11% [7] to 68% [8] after 12–16 weeks with an mean TDI score increase from 2.51 to 4.7 points. Also, OT showed improvement of olfactory function independent of the duration of OD symptoms [9].OT is based on the systematic exposure to four selected odorants (phenyl ethyl alcohol – rose; eucalyptol – eucalyptus; citronellal – lemon; eugenol – cloves), twice a day, for a period of 12 weeks.

However, there is an ongoing debate about the most effective training regimen considering the duration of OT [10–12], adding flavor components to OT [9], repetitions per day [13], and number as well as qualities of odorants used in OT [14]. To address this debate, our aim was to compare OT with an extended number of odors to the classical four-item OT.

The underlying mechanisms of how OT is affecting the odor recognition process are not yet completely understood. Odor processing occurs at several levels in the olfactory system. Odor molecules enter the nose and bind to olfactory receptor neurons (ORN) in the olfactory mucosa [15]. Following activation, the signals reach the olfactory bulb via the axons of the ORN and project further to cortical brain regions and secondary olfactory structures [16]. Disruptions of this fine-orchestrated process lead to OD with either hyposmia (reduced sense of smell), anosmia (no sense of smell), parosmia (qualitatively distorted perception of a smell) and/or phantosmia (perception of smell in the absence of an odor source) [17]. In the case of COVID-19 related OD changes are present at the mucosal level [18]. For example, Bryche et al. [19] showed in a hamster model that two days after SARS-CoV2 infection the main olfactory mucosa was disrupted. For the recovery of this dysfunction, one possible mechanism of OT was suggested by Kim et al. [20]. In a mouse model for OD mRNA analysis were performed showing a stimulation of olfactory receptor expression in the neuroepithelium after OT. The concomitant improvement of olfactory function was shown on a behavioral level by a food-finding test [20]. In humans, OT was associated with an increased number of responses (electro-olfactograms) form the olfactory mucosa [21]. Beyond peripheral changes, OT is also associated with central improvements. For example, hyposmic patients exhibited after OT an increase of grey matter volume in secondary olfactory structures such as the hippocampus and thalamus [22]. In addition, OT improved olfaction over baseline performance in both patients with smell impairment and healthy individuals [23]. The olfactory improvement was associated with, for example, improved cognition as measured by verbal fluency and cognitive tests [24].

In the present pilot study, we focused on the effect of OT in patients with OD (hyposmia and anosmia) and in healthy normosmic participants who completed training with four or seven odors for three months. In order to evaluate the improvement of olfactory recovery, the the “minimal clinically important difference” (MCID) was applied based on Gudziol et al. (2006) [25] and cognitive function was assessed via verbal fluency, MOCA and d2-R tests.

## Materials and methods

This study was approved by the Ethics Committee at the Carl Gustav Carus University Clinic of the TU Dresden (application number: EK 289062019 ) and was conducted following the principles for medical research involving human subjects as described in the declaration of Helsinki [26]. All participants provided written informed consent.

Detailed description of material and methods is provided under S1.

### Study groups and design

In this study 60 normosmic, healthy participants and 40 patients with olfactory dysfunction (OD) were included. From March to November 2021, patients with OD related to COVID-19 or other viral infections presented themselves at the Smell & Taste Clinic of the Department of Otorhinolaryngology at the TU Dresden. During this time period, the SARS-CoV-2 variants Alpha and Delta were dominant [27], revealing a higher prevalence of neurological symptoms such as anosmia, dysosmia, brain fog, depression, delirium, and headache) [28]. Patients received a complete otorhinolaryngological workup including nasal endoscopy. All study participants received a standardized medical interview [29] to record, among others, age, gender, presence of disease, intake of drugs, quantitative and qualitative OD, etiology and duration of OD (Table 1). Qualitative olfactory dysfunction was assessed by an otorhinolaryngologist during a medical interview according to Hummel et al. [30]. In brief, patients are interviewed whether they experienced parosmia and/or phantosmia. If answered in the affirmative, the subjective degree of the olfactory disorders is queried. A score of 0 or 1 is given for each of the following three questions, and the degree of olfactory disorder is sum of the scores. In the first question, the patient is asked whether the qualitative olfactory disorder is experienced either daily (= 1) or not every day (= 0). Then, the patient is asked if the intensity of parosmia or phantosmia is very intense (= 1) or less intense (= 0). Finally, patients are asked whether this symptom has led to secondary effects such as weight loss or major social consequence (= 1). Exclusion criteria were: Pregnancy, smoking (> 5 cigarettes per week), alcohol abuse, neurodegenerative diseases such as Parkinson’s disease and Alzheimer disease, and disorders related to significant olfactory dysfunction, such as severe head trauma. In addition, all participants underwent a nasal endoscopy to exclude OD of other etiologies such as chronic rhinosinusitis.

**Table 1:** Descriptive statistic of the study group

		Normosmic (*n* = 18)	Normosmic + 4-OT (*n* = 18)	Normosmic + 7-OT (*n* = 15)	OD + 4-OT (*n* = 16)	OD + 7-OT (*n* = 17)
Age	Mean	33.2	39.2	41.3	41.0	38.2
	SD	11.0	15.7	13.7	13.1	14.8
OT days	Mean	-	107.9	100.6	109.2	112.1
	SD	-	11.9	25.3	15.8	18.6
Gender (n)	women	13	12	10	10	12
	men	5	6	5	6	5
Quantitative OD (n)	normosmic (no)	16	16	14	4	4
	hyposmic (hy)	2	2	1	10	8
	anosmic (an)	0	0	0	2	5
Qualitative OD (n)	Parosmia	0	0	0	9 (no = 3; hy = 5; an = 1)	11 (no = 2; hy = 7; an = 2)
	Phantosmia	0	0	0	4 (no = 1; hy = 3)	4 (no = 1; hy = 2; an = 1)
Ethiology (n)	post-viral	0	0	0	7	5
	COVID-19	0	0	0	13	15
Duration of disease (n)	< 3 months	-	-	-	2	1
	> 3 months	-	-	-	14	16

n = number, OT = olfactory training, OD = olfactory dysfunction, SD = standard deviation; 4-OT = 4 item olfactory training, 7-OT = 7 item oflactory training

To investigate the role of OT in the olfactory system, we used 4 odorants that have been established for classical OT plus 3 odorants with known receptor activation for the extended OT. Additionally, swabs were taken from the nasal mucosa and cognitive tests were performed. The 4 “classical” odorants were phenyl ethyl alcohol – rose; eucalyptol – eucalyptus; citronellal – lemon; eugenol – cloves. The additional 3 odorants were (L)-(–)-carvone – mint, β-damascenone – stewed apple, salicylic acid benzyl ester – balm. Participants and patients were randomly divided into five groups (*n* = 20, respectively). The first group served as a healthy, normosmic control group and did not train with odors (normosmic). The second group performed a four item OT (normosmic + 4-OT); the third group trained with seven odors (normosmic + 7-OT); the fourth group included patients who trained with four odors (OD + 4-OT), and in the last group patients trained with seven odors (OD + 7-OT). After three months, all participants and patients returned to the ENT clinic for follow-up examination.

From the 100 initially enrolled participants and patients, 16 dropped out before the second appointment (respective numbers are stated in Table 1). Notably, the normosmic, normosmic + 4-OT, and normosmic + 7-OT groups included 5 hyposmic subjects. This is due to the fact that these healthy individuals did not report any impairment of their sense of smell and were not aware of their reduced sense of smell. Normosmic individuals in the patient group (OD + 4-OT and OD + 7-OT) were included because of their qualitative olfactory impairment. Two subjects assigned to the patient group complained about their smell perception after viral infection although showing no qualitative or quantitative impairment.

### Enrollment of patients without OT

To validate the effect of OT per se, a patient group without OT would enhance the validation of the effectiveness of 4-odor and 7-odor OT. Following the guidelines of *The Declaration of Helsinki* new interventions as the 7-odor-item OT should be tested against the “best current proven” intervention [26]. In the position paper from Hummel et al. (2023) [31], OT is the recommended treatment option for patients with olfactory loss of various etiologies. In light of these ethical considerations, we opted not to implement a randomized study group of patients without OT. To address this limitation, we retrospectively included patient data with OD who did no OT from the publications Drews et al. (2022) [32] and Liu et al. (2020) [33]. Patients visited the respective clinics at least twice with either 8 weeks (= 8w; recruitment period August 2012 to February 2013; Drews et al.) or 36 weeks (= 36w; recruitment period from 2008 to 2018; Liu et al.) between both consultations. The description of the study group is provided in the above mentioned publications. All inclucded patients had post-infectious OD. The patient data has been pseudonymised, preventing any conclusions about the identity of the individual.

## Procedure

### Psychophysical testing of olfactory function

Participants and patients were tested twice, before and after three month of OT. Psychophysical testing of olfactory function was performed using the “Sniffin’ Sticks” (Burghart Messtechnik, Holm, Germany) compromising three subsets, namely *T*hreshold, *D*iscrimination, and *I*dentification (TDI). Odors are presented in pen-like odor dispensers. Threshold testing was performed using a triad of pens where one had the target odor, while two other pens were filled with solvent (3-alternative forced choice task, 3-AFC). For testing n-butanol was presented in 16 dilution steps starting from 4% n-butanol (dilution ratio 1:2). Triplets were successively presented to an individual starting from the lowest concentration following a staircase method where two consecutive correct or one incorrect response results in a respective decrease or increase in the subsequently presented concentration. The discrimination test uses the same 3-AFC method as threshold, but each triplet is instead composed of two pens with the same odor and one with a different one. In the identification test, 16 odors are presented, which are each identified from lists of four descriptors. The sum of scores from the three subsets results in a composite TDI score, which allows to diagnose participants with normosmia (> 30.5 points), hyposmia (≤ 30.5 points), or anosmia (< 16.5) [34]. In addition to the standard psychophysical testing, thresholds were assessed for β-damascenone, L-()-carvone, and salicylic acid benzyl ester. The additional three odors were diluted 1:10 starting from 1% slution and threshold testing was performed as previously described in Hummel et al. [3]. Only 8 dilution levels were tested.

In order to evaluate the recovery rate of OT, the MCID was used after Gudziol et al. [25]. The MCID is defined for n-butanol as “probability of subjectively improved odor sensitivity as a function of improving test scores“ [25]. A MCID was set as an increase in composite TDI score of at least 5.5 points or in the threshold subtest (n-butanol) of at least 2.5 points. Accordingly, because of the higher dilution ratio of 1:10 for β-damascenone, L-(–)-carvone, and salicylic acid benzyl ester, the MCID was assumed at a change of 1 point. However, this adaption is providing an estimate.

### Cognitive tests

The Montreal Cognitive Assessment (MOCA) is a screening tool for the onset of a mild cognitive impairment or stronger cognitive dysfunctions [35]. A cut-off value of ≤ 23 out of 30 was implemented to exclude subjects with possible onset of dementia. A score of ≥ 27 points was considered as healthy [36].

The verbal fluency test is a brief test of verbal functioning. Participants are asked to generate as many single words as possible within a semantic category (category fluency) or beginning with a given letter (letter fluency) within 1 min [37].

The d2-R test is a fast test to measure the concentration performance. Subjects are ask to tick in a list with d’s and p’s only the letter d with to lines either above or under the letter within one minute. In doing so, “percentage of errors, concentration performance, errors of omission, and/or errors of commission” were retrieved from this test [38–40].

### Self-reported perception of odors

Participants and patients were asked in their first medical interview to rate in a 7-point scale their olfactory ability) in the following categories: no olfactory ability, very bad, bad, intermediate, good, and very good. In the follow-up visit, participants and patients were asked if there was an improvement of olfactory ability, a change in aroma recognition, and a changed perception of parosmia and phantosmia with following categories: worse, equal, never a problem, slightly improved, clearly improved, and not anymore a problem. All individuals who stated “never a problem” were excluded in the analysis. All patients who performed OT were additionally asked if the perception of odors in general and of the trained odors changed with following categories: less attention to odors, no change, and more attention to odors.

### OT and compliance

In order to verify the adherence of OT, a modified 4-item Morisky scale was adapted [10, 41]. All participants were asked to complete a diary during OT. At the end, a score was assigned according to the proportion of the completed diary, which was composed as follows: Forgotten (no/yes), negligence (no/yes), discontinued (no/yes), complete olfactory diary (no/yes). Therefore, a score ranging from 0 to 4 was reached. Based on median split, a cut-off value of ≥ 2 was set, with 0 and 1 indicating good compliance and 2–4 indicating moderate to low compliance.

### Statistics and data visualization

Statistical analysis was performed with IBM SPSS Statistics (version 28.0.1.0; IBM, Chicago, Ill., USA). In Table 1 we reported the descriptive statistics following the principle intention-to-treat analysis [42]. There we reported all recruited study participants independent of their dropouts. First, group homogeneity was verified for gender, dropouts, age, education years, and compliances score of OT (Morisky score; [41]) among groups. Pearson’s Chi square test showed no significant differences regarding gender distribution and dropouts. One-Way ANOVA followed by Bonferroni correction for multiple comparison yielded no significant differences for age, education years, and Morisky score between groups. Further, independent-samples Mann-Whitney U Test showed no significant differences of the described parameters across dropouts. Therefore, group homogeneity was assumed.

For the comparison to the retrospectively collected data, mean age of the 36w group (66.33 ± 10.06) was significantly higher when compared to all other groups (F_4_ = 21.93).

In the further analysis, only samples are considered which are present in both time points. As a next step, data was checked for normal distribution with Shapiro-Wilk test. For normal distributed data, parametric tests were performed, otherwise non-parametric tests were applied. Time and group effects of olfactory functioning (TDI score), the additional three threshold test (threshold scores), and cognitive tests (verbal fluency test, MOCA test, d2-R test) were analysed by using general linear model followed by Bonferroni post hoc tests for multiple comparisons. In order to compare thresholds displayed as odor concentration, two different test were performed: To analyse a time effect within one threshold test, a related-samples Wilcoxon Signed Rank test was performed. To evaluate an effect between groups, an independent-samples Kruskal-Wallis test was performed followed by Bonferroni post hoc tests for multiple comparison on ranks. Ratings of olfactory impairment and ability, perception and changes of perceived odors, perception of parosmia and phantosmia were analysed at T1 (after OT). For this purpose, an One-Way ANOVA was performed followed by Bonferroni post hoc tests for multiple comparison. In order to examine differences in change over time (T1 – T0), a One-Way ANOVA was performed followed by Bonferroni post hoc tests for multiple comparison.

Level of significance was set at *p* < 0.05 with * *p* < 0.05; ** *p* < 0.01; and *** *p* < 0.001. The correlation plot was implemented in R in RStudio (RStudio for windows, version 2022.07.1, Boston, USA), and all other data was visualized with Graphpad Prism (version 8.4.3; Boston, USA), and shows the data as median ± standard deviation (SD) if not stated differently.

## Results

### After OT the composite TDI score improved significantly in patients with OD

In order to receive an estimate about the beneficial extend of OT, patient data with OT (OD + 4-OT and OD + 7-OT) was compared to patients with OD without OT either 8 weeks or 36 weeks between two visits (S2 FigA-D). A significant increase of the composite TDI score of in both patient groups with OD was visible with *p* ˂ 0.001 after three months but not for patients without OT (after 8 weeks or 36 weeks).

**Fig. 1:**
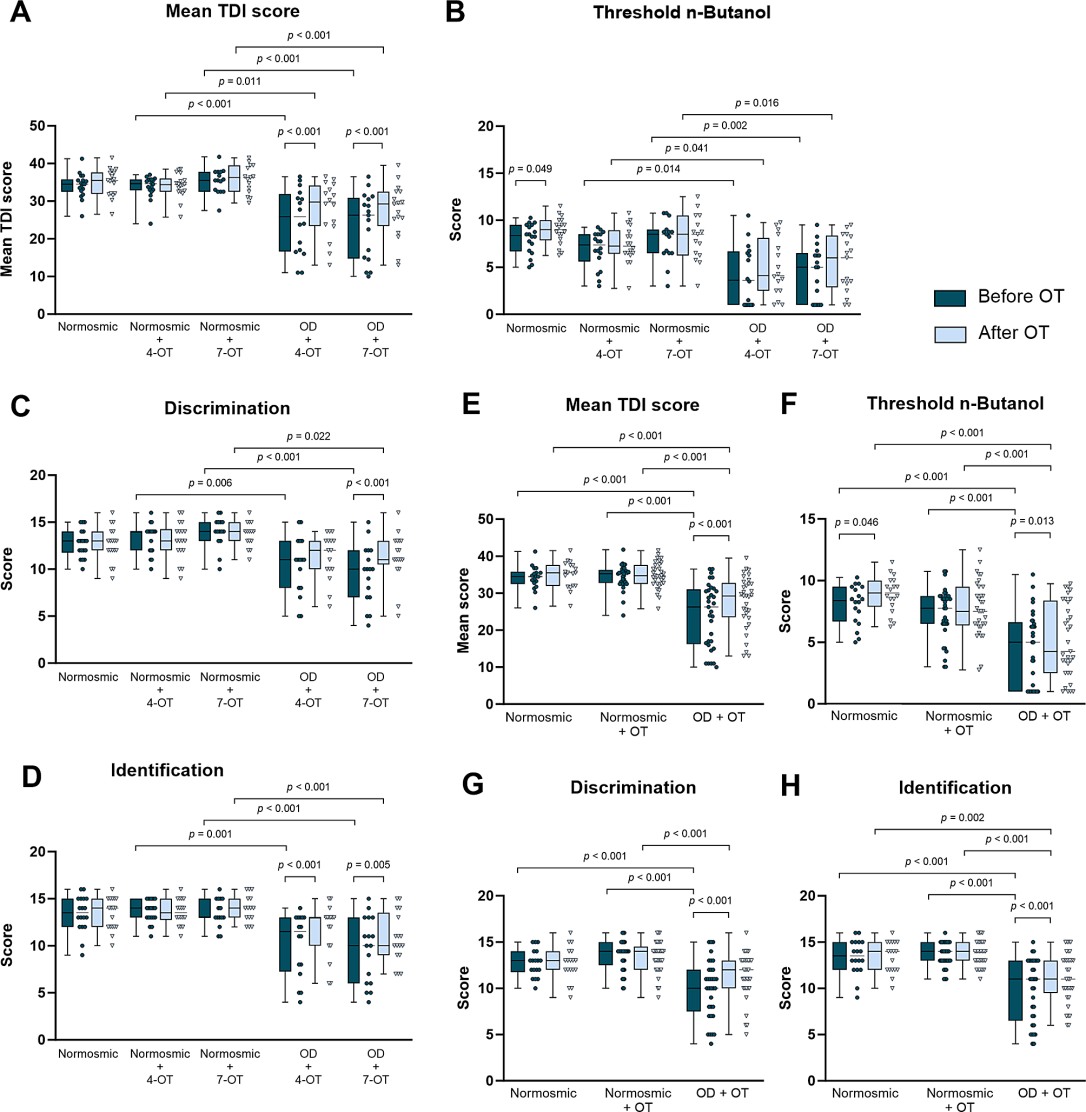
Changes in threshold, discrimination, identification, and combined TDI scores. **A**: After three months olfactory training patients with olfactory dysfunction showed a significant increase of the composite TDI score with *p* ˂ 0.001. **B–C**: Scores of threshold, discrimination and identification tests. Normosmic *n* = 18, normosmic + 4-OT *n* = 18, normosmic + 7-OT *n* = 15, OD + 4-OT *n* = 16, OD + 7-OT *n* = 17. **E–H**: Composite TDI, threshold, discrimination, and identification test with combined normosmic participants with OT and patients with OD and OT (normosmic *n* = 18, normosmic + OT *n* = 33, OD + OT *n* = 33). For better visuality, the significant values between the normosmic group and patients with OD are not displayed (for both time points: A: *p* ≤ 0.002; B, E-H: *p* < 0.001; C: Before OT: normosmic vs. OD + 4-OT *p* < 0.001, normosmic vs. OD + 7-OT non-significant, After OT: non-significant, D: Before OT: *p* ≤ 0.003; After OT: normosmic vs. OD + 4-OT non-significant, normosmic vs. OD + 7-OT *p* = 0.004; G: Before OT: *p* < 0.001, After OT: non-significant). Data is represented as median ± SD. Abbreviations: 4-OT, 4 item olfactory training; 7-OT, 7 item olfactory training; OD, olfactory dysfunction; #, non-significant comparison

Next, patients with OD were compared to normosmics receiving OT or no OT. There, after three months of OT, a significant increase of the composite TDI score of in both patient groups with OD was visible with *p* ˂ 0.001 (Fig. 1A, F_1,79_ = 18.53 for time effect and F_4,79_ = 3.58 for time*group interaction effect). The TDI score increased for OD + 4-OT 3.16 ± 4,46 points and for OD + 4-OT about 3.79 ± 3.79 points. This improvement was mainly related to an increase of identification score (Fig. 1B-D, p ˂ 0.001 and *p* ˂ 0.01). Despite OT, the TDI score of the patient groups remained significantly lower when compared to the healthy controls (*p* ˂ 0.001). No differences in the mean TDI score were detected between four and seven item OT groups both in the healthy controls as well as in the patient groups. Therefore, we merged the data and displayed combined groups to emphasize the effect of OT and to increase the sample size for statistical analysis (normosmic, normosmic + OT, and OD + OT; Fig. 1E-H). The mean increase of the patient’s TDI score was 3.48 ± 4.21 higher after OT.

**Fig. 2:**
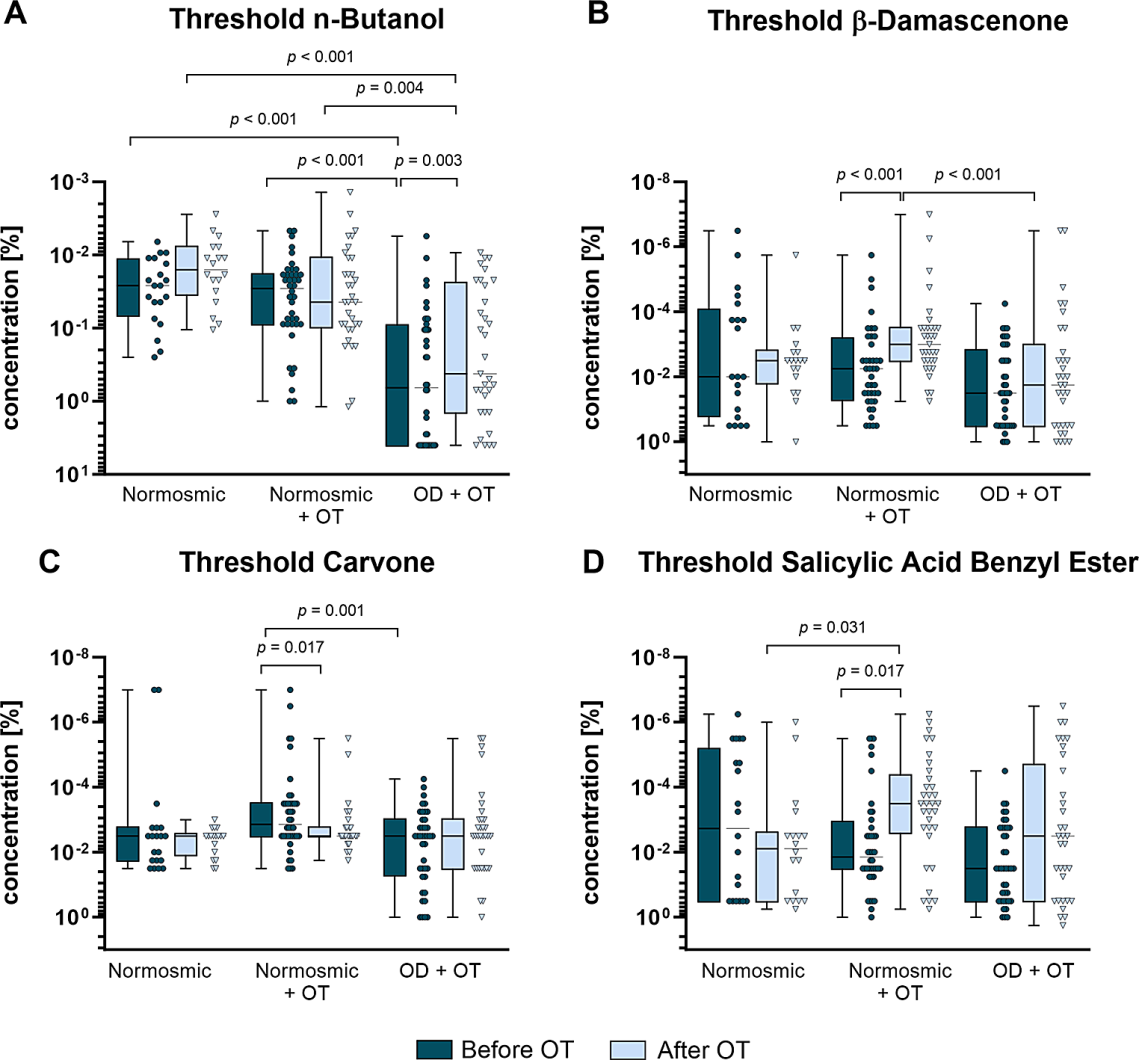
Detected odour threshold concentrations of n-butanol (**A**), β-damascenone (**B**), carvone (**C**), and salicylic acid benzyl ester (**D**). n-Butanol was diluted in steps of 1:2 starting with a 4% odor concentration and was tested by using a 16- staircase [3]. The remaining three odors were diluted in steps of 1:10 starting with a 1% dor concentration. Threshold testing were performed in a 8-way staircase. Group size: Normosmic *n* = 18, normosmic + OT *n* = 33, OD + OT *n* = 33. Data is represented as median ± SD. Abbreviations: OT, olfactory training; OD, olfactory dysfunction; #, non-significant comparison

The results for the odor threshold concentration of β-damascenone, L-(–)-carvone, and salicylic acid benzyl ester are shown as odor concentration in % (Fig. 2). In healthy participants OT was asscoiated with significantly higher sensitivity towards β-damascenone (*p* < 0.001, Fig. 2B) and salicylic acid benzyl ester (*p* < 0.01, Fig. 2D) and towards n-butanol in patients with OD (*p* < 0.01, Fig. 2A). A tendency of this effect was also observed in OD + OT group towards salicylic acid benzyl ester (Fig. 2D). Hence, the results indicate a beneficial effect of OT per se regarding specific anosmia towards β-damascenone and salicylic acid benzyl ester with a higher improvement in healthy participants.

This finding is underlined by the number of healthy participants and patients who improved in the MCID, which reflects the OT-mediated recovery rate (Table 2). The cut-off value emerged from the quantitative relationship between test scores and percept of olfactory function which was adapted after Gudziol et al. [25]. Normosmics with OT revealed the highest number of participants which improved in the MCID in the threshold test for β-damascenone (*n* = 21) followed by salicylic acid benzyl ester (*n* = 15). The largest number of patients showing a MCID was found for salicylic acid benzyl ester (*n* = 14), followed by β-damascenone, and L-(–)-carvone) (*n* = 10). Summarizing, OT led to an improvement of the TDI score in approximately one third of patients with OD.

**Table 2:** Number of individuals reaching the MCID in threshold testing and TDI score

		Improvement of threshold score				Improvement of TDI score ≥ 5.5
		≥ 2.5	≥ 1			
		n-butanol	β-damascenone	carvone	salicylic acid benzyl ester	
Normosmic (*n* = 18)	*n*	3	6	3	3	2
	in %	16.67	33.33	16.67	16.67	11.11
	Mean	3.58	2.04	1.08	3.42	6.5
	SD	0.76	0.84	0.14	1.38	0.35
	Minimum	2.75	1	1	2.5	6.25
	Maximum	4.25	3	1.25	5	6.75
Normosmic + OT (*n* = 33)	*n*	4	21	4	15	2
	in %	12.12	63.64	12.12	45.45	6.06
	Mean	4.19	1.83	1.56	2.53	6.38
	SD	1.95	0.953	0.43	1.04	0.53
	Minimum	2.75	1	1	1	6
	Maximum	7	4.75	2	5	6.75
OD + OT (*n* = 33)	*n*	5	9	10	14	12
	in %	15.15	27.27	30.30	42.42	36.36
	Mean	3.3	2.92	1.88	3.07	8.02
	SD	1.12	1.26	0.91	1.21	2.83
	Minimum	2.5	1.5	1	1.25	5.5
	Maximum	5.25	5	3.75	5.5	12.75

OT = olfactory training, OD = olfactory dysfunction, n = number, SD = standard deviation

### OT ameliorated MOCA score in patients with OD

**Fig. 3:**
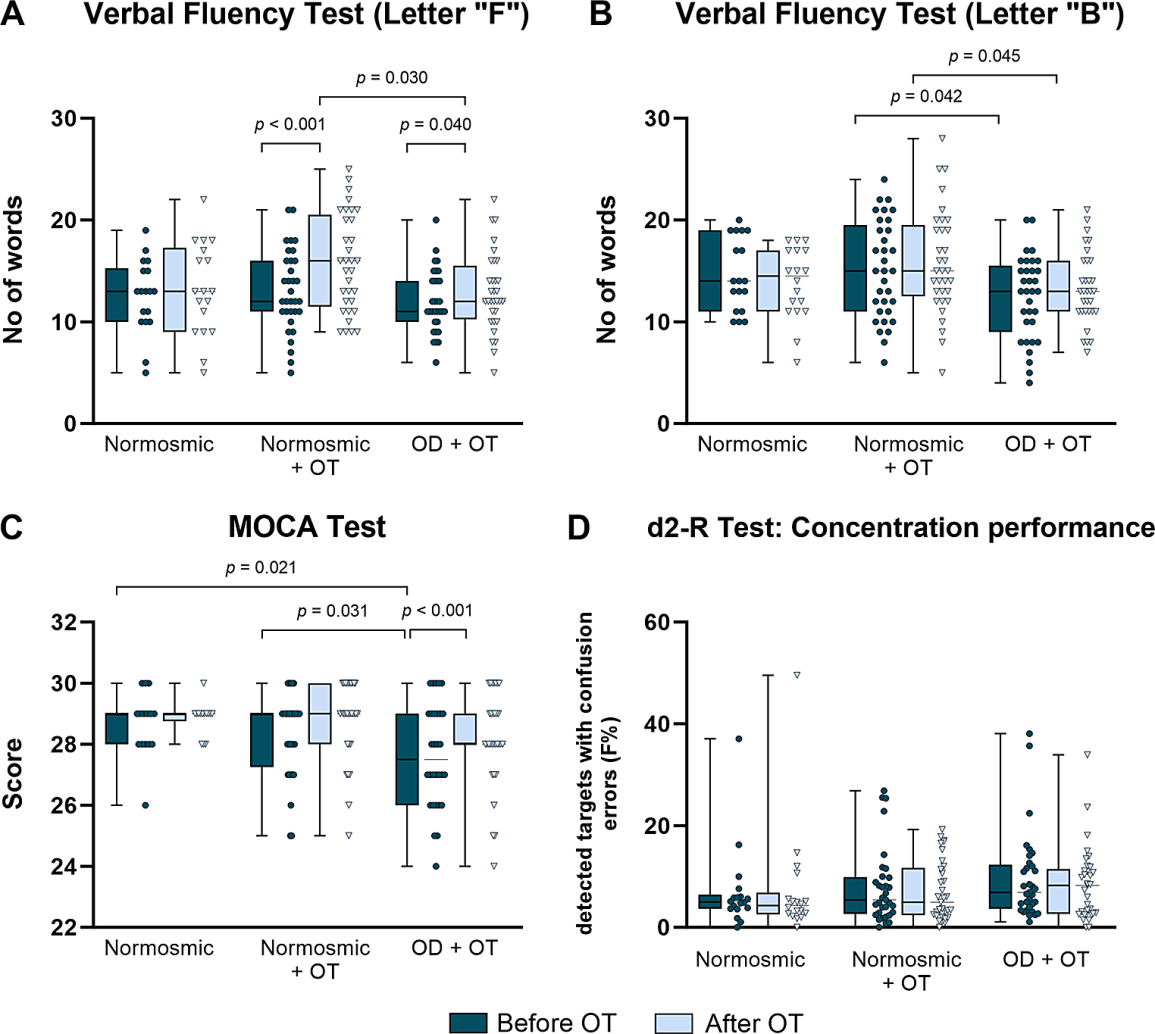
Cognitive abilities. **A**,** B**: Verbal fluency tests staring with letter “F” and “B”. OT led to a significant increase of verbal fluency in normosmic + OT with *p* < 0.01 and OD + OT with *p* < 0.001 (Before OT: normosmic *n* = 18, normosmic + OT *n* = 33, OD + OT *n* = 33; After OT: normosmic *n* = 18, normosmic + OT *n* = 33, OD + OT *n* = 32.) **C**: Montreal cognitive assessment (MOCA) test. OT led in the group OD + OT to a significant increase of score points with *p* < 0.001. Participants with ≤ 23 were excluded from this test. (Before OT: normosmic *n* = 15, normosmic + OT *n* = 32, OD + OT *n* = 30; After: normosmic *n* = 14, normosmic + OT *n* = 32, OD + OT *n* = 31). **D**: d2-R test. A positive effect of olfactory training on the concentration performance was absent (Normosmic *n* = 18, normosmic + OT *n* = 33, OD + OT *n* = 33). Data is represented as median ± SD. Non-significant comparison are not shown in the figure. Abbreviations: OT, olfactory training; OD, olfactory dysfunction

OT led to a significant increase of the verbal fluency in both groups (Fig. 3A + 3B; F_1,80_ = 15.20 for time effect, normosmics + OT *p* < 0.001; OD + OT *p* < 0.019). Then, in the MOCA test all groups exhibited a mean MOCA score ≥ 27 points and were considered as healthy regarding cognitive impairment. But, OT improved significantly the mean MOCA score in patients with OD from 27.22 ± 2.08 points to 28.16 ± 1.57 points (Fig. 3C; F_1,74_ = 7.22 for time effect, *p* < 0.001). However, this cognitive improvement was not related to better concentration performance (Fig. 3D). To sum it up, in OD patients OT was associated with small but significant improvement of cognitive abilities.

### Effects of three month OT

In patients with OD, OT was asscoaited with improvement of the composite TDI score (S3 Fig A, effect of the factor “group”: *F*_2_ = 7.18, *p* < 0.001; *p* < 0.05 vs. normosmic and *p* < 0.01 vs. normosmic with OT). Additionally, an improvement was observed in the threshold subset for n-butanol with *p* < 0.05 when compared to the other two groups (S3 Fig B, *F*_2_ = 5.72 for between group effect, *p* = 0.005). In the other collected data (S3 Fig C – I), OT was mainly associated with a training effect within the groups.

### Ratings of olfactory function increased after OT

**Fig. 4:**
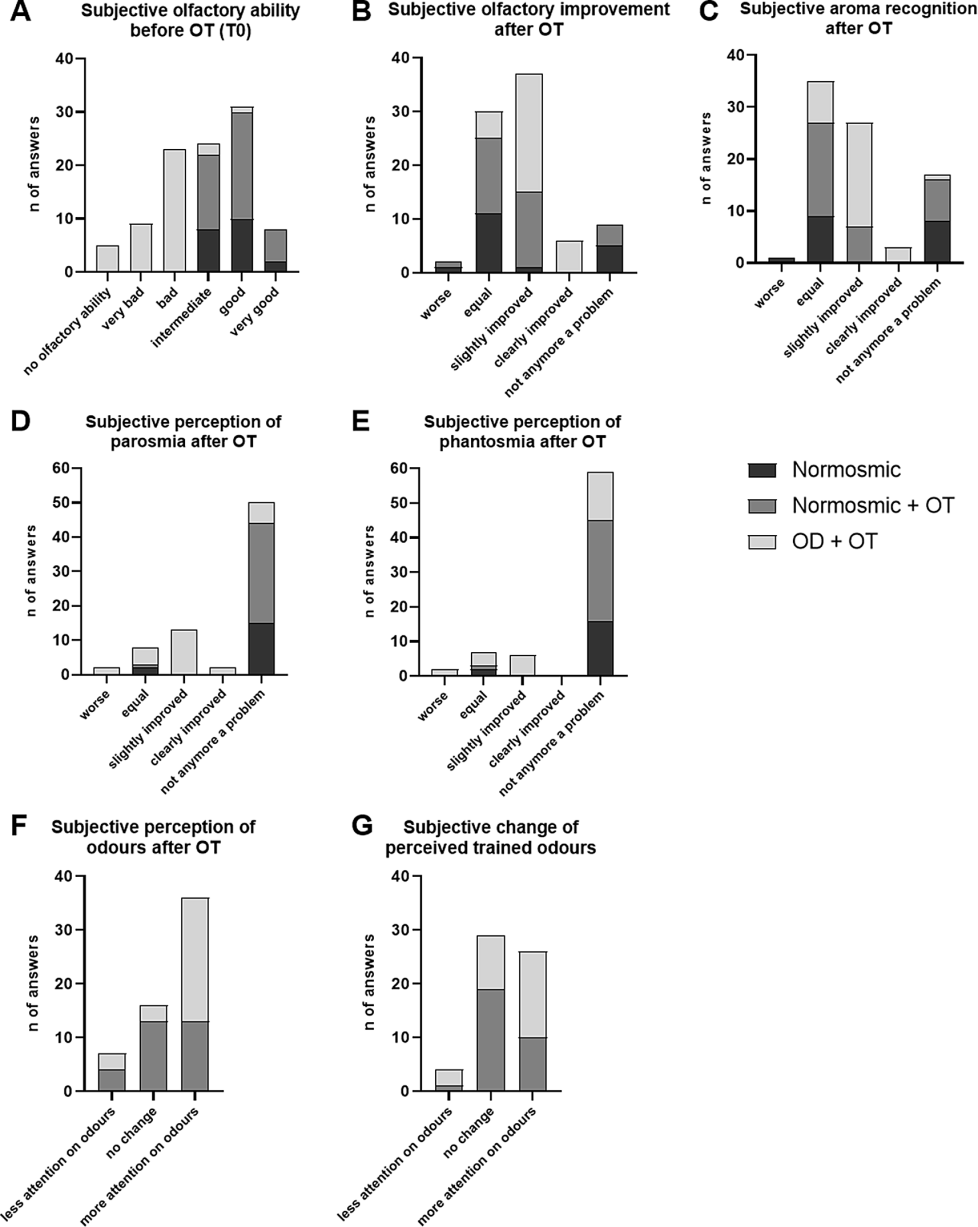
Ratings about olfactory ability and olfactory function. **A**: Subjective olfactory ability was assessed with in a standardized medical interview. After OT, subjective olfactory improvement (**B**), aroma recognition (**C**), perception of parosmia (**D**) and phantosmia (**E**) was assessed in a 6-point Likert-type scale. Additional ratings regarding the perception of odors after OT (**F**) and the trained odors (**G**) was gathered. Data is presented as amount of answer for each question. For ratings about olfactory ability from patients with qualitative OD, see S3 Fig

Reported olfactory function increased after OT, especially in the patient group. Before OT, all patients (*n* = 40) reported olfactory impairment; 23 patients out of 40 reported to have a bad, 9 a very bad, and 5 no olfactory ability (Fig. 4A). After OT, 22 patients out of 34 reported a slight improvement of the olfactory ability (Fig. 4B) coupled with a slight improvement regarding aroma recognition (*n* = 20; Fig. 4C), parosmic sensations (*n* = 13; Fig. 4D), and phantosmia (*n* = 6; Fig. 4E). Furthermore, OT led in the patient group to more attention on the perception of odors (*n* = 23; Fig. 4F) and also on the trained odors which were β-damascenone, carvone, and salicylic acid benzyl ester (*n* = 16; Fig. 4G). A further description of the perception of olfactory functions after OT for parosmia and phantosmia is given in S4 Fig. The improvement of rated olfactory function in OD patients was more pronounced compared to normosmics with OT (*p* < 0.001) (see Table 3). However, no significant group effect was obeserved for the perception of parosmia and phantosmia.

**Table 3:** Ratings about olfactory function after OT (T1)

	Independent-Samples Kruskal-Wallis Test Summary		Bonferroni test for multiple comparison		
	F, DF	*p*-value	Normosmic vs. Normosmic + OT	Normosmic vs. OD + OT	Normosmic + OT vs. OD + OT
Subjective olfactory ability before OT	42.76 2.00	< 0.001	ns	< 0.001	< 0.001
Subjective olfactory improvement after OT	28.54 2	< 0.001	ns	< 0.001	< 0.001
Subjective aroma recognition after OT	18.26 2	< 0.001	ns	< 0.001	< 0.001
Subjective perception of parosmia after OT	3.02 2.00	ns	-	-	-
Subjective perception of phantosmia after OT	1.46 2.00	ns	-	-	-
	**Independent-Samples Mann-Whitney U Test.** ***p*** **-value**				
Subjective perception of odors after OT		0.045			
Subjective change of perceived trained odors		ns	-	-	-

F = F-value, DF = degree of freedom, OT = olfactory training, OD = olfactory dysfunction, ns, not significant

### TDI score ≥ 5.5 correlates with the improvement of rated olfactory function

In order to evaluate correlations between all observed parameters, a multidimensional correlation matrix was implemented (Fig S4; S5 Table). In the following, only significant correlations were considered. Naturally, the change in the TDI score correlated strongly positive with the change of TDI score ≥ 5.5 (= MCID, *p* < 0.001) and the change of n-butanol threshold (*p* < 0.001). Regarding the difference and improvement in cognitive tests which are MOCA, d2-R, and verbal fluency tests, there were no significant correlations to the change of TDI score or the change of threshold scores (β-damascenone, L-(–)-carvone, and salicylic acid benzyl ester). On the other hand, both, the improvement after OT in TDI score and in the four threshold scores correlated with different parameters of ratings of olfactory function. For example, the improvement of the threshold for salicylic acid benzyl ester correlated with the rated olfactory improvement (*p* < 0.05). Interestingly, the TDI score ‘≥ 5.5 points’, but not the TDI score per se, correlated positively with the perception of parosmia (*r*^2^ = 0.62, *p* < 0.01). This effect mainly relied on the report from 6 out of 10 patients with OD indicating a clear improvement of the perception of parosmia after OT. To sum it up, the rated improvement of olfactory function correlated with the change of measured olfactory function. A slight improvement of the perception of parosmia correlated only with the MCID of the TDI score.

## Discussion

The main findings of the pilot study were that, firstly, OT was concomitant with the improvement of the composite TDI score of 3.48 points in patients with OD. However, OT with seven odors had no additional effects on the improvement of olfactory function. Secondly, for OD patients this improvement is reflected in the TDI score-related MCID (≥ 5.5 points; a measure for the olfactory recovery) and for the salicylic benzyl ester threshold-related MCID (≥ 1 point). Furthermore, OT improved parameters of cognitive function of patients as assessed with the MOCA test and rated olfactory improvement or perception of parosmia.

### Four item vs. seven-item OT

One idea for using a seven-item OT was that presenting a wider range of odors would enhance the effect of OT by activating a more diverse set of ORN [43]. In addition, the augmented training was thought to be more beneficial for OD patients. However, neither one of these effects was observed after three-month OT with seven odors. A previous study by Pires et al. obtained analogous results in patients with persistent OD caused by COVID-19 [44]. According to the authors, eight representatives of the odorous space regarding citric, floral, aromatic, spicy, minty, sweet, and woody aromas were added to essential oils of citronella, mint, vanilla, and cedar wood. After four weeks of OT with the extended set of odors, the odor identification test revealed no increased recovery when compared to a four-item OT [44]. This result is in line with the present findings where four odorants achieved comparable results as a seven-item OT. Another approach to enhance the effectiveness of OT was to train more frequently per day (four times) [13]. Oleszkiewicz et al. showed that the intense OT did not result in an improvement of olfactory function when compared to a regular OT regimen. This outcome supports the present results that OT with four odors appears to be enough to support recovery of olfactory function.

Interestingly, considering a more effective treatment towards parosmia, Altundag et al. [45] established a modified version of OT. Altundag and colleagues extended the duration of OT to nine months and changed the odors three times during the training period [46]. They demonstrated that patients changing the odors every three months scored significantly higher in odor identification and discrimination when compared to a group training with the same set of odors for 9 months. The improvement became more pronounced the longer the training. In the presently studied population, we were unable to detect a similar effect. However, there is a considerable difference in terms of the length of therapy: In the present study, OT was carried out for three months; in the previously referred publications, the duration of training varied from four weeks [44] to 9 months [14]. Thus, one possible explanation of varying outcomes might be the duration of training. Possibly, there would have been an effect from the seven-item OT if the training had been extended to 6 or 9 months. Furthermore, it might be also possible that the number of odorants which are used for training have less impact on the outcome of OT than the duration period. Therefore, we hypothesize that, assuming consistent adherence to OT [10], possibly the length of OT is more crucial than the intensity of OT.

### Impact of OT in central or peripheral processes

The results from the present study suggest that OT led to a significant increase in cognition assessed by a verbal fluency test and the MOCA test. Of particular interest is the observed significant increase in cognition in the patient group depicted by the MOCA test. Currently, it is not entirely clear which changes the OT will entail (“Training-Based Enhancement” in [47]). One current opinion assumes central cognitive changes. In the literature it is reported that OT led to improved olfactory function which was accompanied by enhanced cognition and changes in neurological structures and connections [48]. In the review by Vance and colleagues it was summarized that the changes included the attenuation of cognitive decline [24], subjective well-being, and improved verbal function [49]. This concept of OT having an effect on cognitive components is further supported by Olofsson et al. [50]. This work investigated whether olfactory-related memory training leads to a carry-over to visual memory tasks that have not been trained. The authors showed that olfactory but not visual training produced transfer effects. Hence, Olofsson and colleagues stated that smell-based memory training may result in increased connectivity of the sensory brain areas compared to the visual paradigm [50]. One potential underlying mechanism for this finding is that olfactory function has a relatively direct effect on memory encoding regions. This might lead to a greater overlap with the neural networks involved in memory encoding and thereby facilitating transfer effects [51]. Recent work on changes in hippocampal volume (involved in memory processing [52]) in relation to OT supports these assumptions. Hähner et al. [53] showed that OT had a beneficial effect on the hippocampus by increasing the cortical thickness. However, the olfactory bulb volume was not changed. On the other hand, other studies could show an effect of OT on the olfactory bulb. For instance, the olfactory bulb volume increased after four month OT in healthy adults [54] and after six month of OT for patients with idiopathic olfactory loss [55]. Interestingly, OT appears to have an impact on memory which may allow a new therapeutic use of OT for memory deficits.

The effectiveness of OT depends not only on alterations in memory abilities, also peripheral changes are described. Kim et al. [20] showed a stimulation of the olfactory receptor expression in the neuroepithelium of mice after OT. Consistent with this finding, the same group described one year later in mice that olfactory regeneration most likely began with an early increase in ORN expression [56]. This evidence that OT leads to changes at the level of olfactory mucosa is also supported by a previous work in humans [21]. Patients with OD received four to six months OT accompanied by electro-olfactogram recordings derived from the olfactory mucosa. As patients with post-infectious OD (anosmia) reveal reduced numbers of olfactory receptor neurons [57], lower numbers of electro-olfactograms were obtained when compared to the control group without OT. In contrast, after OT an increase of electrical activity at the level of olfactory epithelium was detected in patients [21]. Thus, it was assumed that the concomitant increase in electro-olfactogram responses and OT led to an increase in expression of olfactory receptors or an increased number of ORN [21]. In order to contextualize our own study in this regard, we took swabs from the nasal mucosa to gain insights if OT leads to altered expression of respective olfactory receptors. Still, the present study indicates that OT might have an impact on central processing, which is suggested by the significant increase of one point in the MOCA test after three months of OT.

### Limitation of the study

The study has some limitations. First, the number of participants starting with *n* = 20 per group constitutes a relatively small sample size to evaluate the effects of adding additional odors to the OT. However, as all the control groups had same group sizes with equal gender, age and education distributions, the outcome appears to be valid as a prospective and pilot study. In addition, other than previous work, the present study had a control group which adds significant value to the study despite the relatively small sample size per group. Further, in order to increase the sample size of the patient groups, hyposmic and anosmic patients were combined. This results in heterogenous groups with different baseline TDI scores which may result in different outcomes. Second, a sixth group is missing only including patients without OT. This group could further validate the effectivness of 4-odor and 7-odor OT. Schepens et al. could show that OT in COVID-19 patients improved psychophysical olfactory function when compared to COVID-19 cohort without OT [58]. However, this effect was not dependend to the adherence to OT. In the present study ethical considerations precluded the enrollment of a patient group without receiving treatment. Summarized in a review by Pieniak et al. (2022) [59], a shorter period from disease onset to OT initiation is positively associated with better TDI scores. The MCID was higher for patients with an olfactory loss less than one year prior to OT initiation than for patients with a prolonged olfactory loss (63% vs. 19%, respectively). In addition, OT is cost-efficient and safe treatment, which is the recommended treatment option for patients with olfactory loss of various etiologies [31]. Third, studies conducted at home commonly show problems with adherence to the training regimen. To address this concern, a modified Morisky scale was assessed, which was evaluated with an olfactory diary. As the Chi Square test did not show significant differences between the groups, it can be assumed that compliance to OT was comparable between groups. Fourth, in the normosmic group hyposmic individuals were present. However, these participants claimed to have a normal sense of smell with no altered perception or distortions. Additionally, they did not present themselves as patients which did not classify them as post-viral patients. Excluding the five individuals did not significantly change the results. In order to increase the sample size these individuals were included. Fifth, a bias might be introduced in the threshold testing as the threshold score in the normosmic group was significantly higher in the second appointment. However, this fluctuation in olfactory thresholds vary considerably among individuals and day-to-day fluctuations within the same individual [60, 61]. Therefore, a fluctuation lower than 2.5 points is plausible which may be affected by the testing methodology [60]. Lastly, a direct adaption of the MCID from n-butanol with a cut-off value of ≥ 2.5 to salicylic acid benzyl ester, β-damascenone, and L-(–)-carvone (cut-off value ≥ 1) should evaluated cautiously. The MCID was determined for n-butanol and reflects a relationship between olfactory percept and improvement of test scores. As the threshold steps for salicylic acid benzyl ester, β-damascenone, and L-(–)-carvone were adjusted, the underlying logistic function should be confirmed. However, the MCID provides an important and reproducible measure of OT.

## Conclusion

In summary, the present study showed that OT over a period of three months with four or seven odors is associated with improvement in olfactory performance for patients with olfactory dysfunction. However, adding more odors to the training regimen appears to be of little or no additional benefit. Importantly the present results support the notion that OT is associated with improvement of cognitive functions.

## Electronic supplementary material

Below is the link to the electronic supplementary material.

Supplementary Material 1

Supplementary Material 2

## Data Availability

All raw data and coding sections can be provided on request. Please contact the corresponding author Nicole Power Guerra, nicole.powerguerra@ukdd.de.

## References

[1] Gerkin RC, Ohla K, Veldhuizen MG, Joseph PV, Kelly CE, Bakke AJ et al (2021) Recent smell loss is the best predictor of COVID-19 among individuals with recent respiratory symptoms. Chem Senses 46(bjaa081):1–12 10.1093/chemse/bjaa081 PMC7799216 33367502

[2] Coelho DH, Reiter ER, Budd SG, Shin Y, Kons ZA, Costanzo RM (2021) Quality of life and safety impact of COVID-19 associated smell and taste disturbances. Am J Otolaryngol 42(4):103001 33773440 10.1016/j.amjoto.2021.103001 PMC7983361

[3] Hummel T, Sekinger B, Wolf SR, Pauli E, Kobal G (1997) Sniffin’ sticks’: olfactory performance assessed by the combined testing of odor identification, odor discrimination and olfactory threshold. Chem Senses 22(1):39–52 9056084 10.1093/chemse/22.1.39

[4] Hwang SH, Kim SW, Basurrah MA, Kim DH (2023) The efficacy of olfactory training as a treatment for olfactory disorders caused by Coronavirus Disease-2019: a systematic review and Meta-analysis. Am J Rhinol Allergy 37(4):495–501 36635974 10.1177/19458924221150977

[5] Hummel T, Whitcroft KL, Andrews P, Altundag A, Cinghi C, Costanzo RM et al (2017) Position paper on olfactory dysfunction. Rhin 54(26):1–30 10.4193/Rhino16.248 29528615

[6] Hopkins C, Alanin M, Philpott C, Harries P, Whitcroft K, Qureishi A et al (2021) Management of new onset loss of sense of smell during the COVID-19 pandemic - BRS Consensus guidelines. Clin Otolaryngol 46(1):16–22 32854169 10.1111/coa.13636 PMC7461026

[7] Fleiner F, Lau L, Göktas Ö (2012) Active olfactory training for the treatment of smelling disorders. Ear Nose Throat J 91(5):198–203 22614554 10.1177/014556131209100508

[8] Konstantinidis I, Tsakiropoulou E, Bekiaridou P, Kazantzidou C, Constantinidis J (2013) Use of olfactory training in post-traumatic and postinfectious olfactory dysfunction. Laryngoscope 123(12):E85–90 24114690 10.1002/lary.24390

[9] Besser G, Oswald MM, Liu DT, Renner B, Mueller CA (2020) Flavor education and training in olfactory dysfunction: a pilot study. Eur Arch Otorhinolaryngol. ; 277(7):1987–94. Available from: URL: https://pubmed.ncbi.nlm.nih.gov/32248300/10.1007/s00405-020-05950-8 PMC7286942 32248300

[10] Saatci O, Altundag A, Duz OA, Hummel T (2020) Olfactory training ball improves adherence and olfactory outcomes in post-infectious olfactory dysfunction. Eur Arch Otorhinolaryngol 277(7):2125–2132 32246254 10.1007/s00405-020-05939-3

[11] Konstantinidis I, Tsakiropoulou E, Constantinidis J (2016) Long term effects of olfactory training in patients with post-infectious olfactory loss. Rhin 54(2):170–175 10.4193/Rhino15.264 27017331

[12] Qiao X-F, Bai Y-H, Wang G-P, Li X, Zheng W (1992) Clinical effects of two combinations of olfactory agents on olfactory dysfunction after upper respiratory tract infection during olfactory training. Rev Assoc Med Bras 2020; 66(1):18–24 10.1590/1806-9282.66.1.18 32130376

[13] Oleszkiewicz A, Bottesi L, Pieniak M, Fujita S, Krasteva N, Nelles G et al (2022) Olfactory training with Aromastics: olfactory and cognitive effects. Eur Arch Otorhinolaryngol 279(1):225–232 33864109 10.1007/s00405-021-06810-9 PMC8051546

[14] Altundag A, Cayonu M, Kayabasoglu G, Salihoglu M, Tekeli H, Saglam O et al (2015) Modified olfactory training in patients with postinfectious olfactory loss. Laryngoscope 125(8):1763–1766 26031472 10.1002/lary.25245

[15] Manzini I, Schild D, Di Natale C (2022) Principles of odor coding in vertebrates and artificial chemosensory systems. Physiol Rev. ; 102(1):61–154. Available from: URL: https://pubmed.ncbi.nlm.nih.gov/34254835/10.1152/physrev.00036.2020 34254835

[16] Hummel T, Welge-Lüssen A (2006) Taste and smell: an update. Karger, Basel. (vol 63)

[17] Hummel T, Power Guerra N, Gunder N, Hähner A, Menzel S, Laryngorhinootologie (2023) ; 102(S 01):S67–S92 10.1055/a-1957-3267 PMC10184680 37130532

[18] Butowt R, Bilinska K, von Bartheld CS (2023) Olfactory dysfunction in COVID-19: new insights into the underlying mechanisms. Trends Neurosci. ; 46(1):75–90. Available from: URL: https://www.cell.com/trends/neurosciences/fulltext/S0166-2236(22)00234-X 10.1016/j.tins.2022.11.003 PMC9666374 36470705

[19] Bryche B, St Albin A, Murri S, Lacôte S, Pulido C, Ar Gouilh M et al (2020) Massive transient damage of the olfactory epithelium associated with infection of sustentacular cells by SARS-CoV-2 in golden Syrian hamsters. Brain Behav Immun. ; 89:579–86. Available from: URL: https://pubmed.ncbi.nlm.nih.gov/32629042/10.1016/j.bbi.2020.06.032 PMC7332942 32629042

[20] Kim B-Y, Park JY, Kim EJ, Kim BG, Kim SW, Kim SW (2019) The neuroplastic effect of olfactory training to the recovery of olfactory system in mouse model. Int Forum Allergy Rhinol. ; 9(7):715–23. Available from: URL: https://pubmed.ncbi.nlm.nih.gov/30793525/10.1002/alr.22320 PMC6767412 30793525

[21] Hummel T, Stupka G, Haehner A, Poletti SC (2018) Olfactory training changes electrophysiological responses at the level of the olfactory epithelium. Rhinology 56(4):330–335 30076701 10.4193/Rhin17.163

[22] Gellrich J, Han P, Manesse C, Betz A, Junghanns A, Raue C et al (2018) Brain volume changes in hyposmic patients before and after olfactory training. Laryngoscope. ; 128(7):1531–6. Available from: URL: https://pubmed.ncbi.nlm.nih.gov/29238983/10.1002/lary.27045 29238983

[23] Vance DE, Del Bene VA, Kamath V, Frank JS, Billings R, Cho D-Y et al (2024) Does Olfactory Training Improve Brain Function and Cognition? A Systematic Review. Neuropsychol Rev. ; 34(1):155–91. Available from: URL: https://link.springer.com/article/10.1007/s11065-022-09573-0 10.1007/s11065-022-09573-0 PMC9891899 36725781

[24] Oleszkiewicz A, Abriat A, Doelz G, Azema E, Hummel T (2021) Beyond olfaction: beneficial effects of olfactory training extend to aging-related cognitive decline. Behav Neurosci 135(6):732–740 34110862 10.1037/bne0000478

[25] Gudziol V, Lötsch J, Hähner A, Zahnert T, Hummel T (2006) Clinical significance of results from olfactory testing. Laryngoscope 116(10):1858–1863 17003712 10.1097/01.mlg.0000234915.51189.cb

[26] World Medical Association Declaration of Helsinki (2001) Ethical principles for medical research involving human subjects. Bull World Health Organ 79(4):373–374 11357217 PMC2566407

[27] Robert Koch Institut SARS-CoV-2 Varianten in Deutschland: Daten aus der integrierten genomischen Surveillance von SARS-CoV-2; 2024 [cited 2024 Jan 5]. Available from: URL: https://public.data.rki.de/t/public/views/IGS_Dashboard/DashboardVOC?%3Aembed=y&%3AisGuestRedirectFromVizportal=y

[28] Canas LS, Molteni E, Deng J, Sudre CH, Murray B, Kerfoot E et al (2023) Profiling post-COVID-19 condition across different variants of SARS-CoV-2: a prospective longitudinal study in unvaccinated wild-type, unvaccinated alpha-variant, and vaccinated delta-variant populations. The Lancet Digital Health. ; 5(7):e421-e434. Available from: URL: https://www.thelancet.com/journals/landig/article/PIIS2589-7500%2823%2900056-0/fulltext 10.1016/S2589-7500(23)00056-0 PMC10187990 37202336

[29] Welge-Luessen A, Hummel T (eds) (2013) Management of smell and taste disorders. [Thieme], Stuttgart

[30] Hummel T, Hummel C, Welge-Luessen A (2013) Assessment of Olfaction and Gustation. In: Welge-Luessen A, Hummel T (eds) Management of smell and taste disorders. [Thieme], Stuttgart, pp 58–75

[31] Whitcroft KL, Altundag A, Balungwe P, Boscolo-Rizzo P, Douglas R, Enecilla MLB et al (2023) Position paper on olfactory dysfunction: Rhin 2023. 10.4193/Rhin22.483 37454287

[32] Drews T, Hummel T, Rochlitzer B, Hauswald B, Hähner A (2022) Acupuncture is associated with a positive effect on odour discrimination in patients with postinfectious smell loss-a controlled prospective study. Eur Arch Otorhinolaryngol 279(3):1329–1334 34032906 10.1007/s00405-021-06872-9 PMC8897321

[33] Liu DT, Pellegrino R, Sabha M, Aytug A, Damm M, Poletti SC et al (2020) Factors associated with relevant olfactory recovery after olfactory training: a retrospective study including 601 participants. Rhin. ; 0(0):0. Available from: URL: https://pubmed.ncbi.nlm.nih.gov/32901616/10.4193/Rhin20-262 33544097

[34] Oleszkiewicz A, Schriever VA, Croy I, Hähner A, Hummel T (2019) Updated Sniffin’ Sticks normative data based on an extended sample of 9139 subjects. Eur Arch Otorhinolaryngol. ; 276(3):719–28. Available from: URL: https://link.springer.com/article/10.1007/s00405-018-5248-1 10.1007/s00405-018-5248-1 PMC6411676 30554358

[35] Nasreddine ZS, Phillips NA, Bédirian V, Charbonneau S, Whitehead V, Collin I et al (2005) The Montreal Cognitive Assessment, MoCA: a brief screening tool for mild cognitive impairment. J Am Geriatr Soc. ; 53(4):695–9. Available from: URL: https://pubmed.ncbi.nlm.nih.gov/15817019/10.1111/j.1532-5415.2005.53221.x 15817019

[36] Thomann AE, Berres M, Goettel N, Steiner LA, Monsch AU (2020) Enhanced diagnostic accuracy for neurocognitive disorders: a revised cut-off approach for the Montreal Cognitive Assessment. Alz Res Therapy. ; 12(1):39. Available from: URL: https://alzres.biomedcentral.com/articles/10.1186/s13195-020-00603-8 10.1186/s13195-020-00603-8 PMC7140337 32264975

[37] Shao Z, Janse E, Visser K, Meyer AS (2014) What do verbal fluency tasks measure? Predictors of verbal fluency performance in older adults. Front. Psychol. ; 5:772. Available from: URL: https://www.frontiersin.org/articles/10.3389/fpsyg.2014.00772/full 10.3389/fpsyg.2014.00772 PMC4106453 25101034

[38] Brickenkamp R (1962) Test d2: aufmerksamkeits-belastungs-test. C. J. Hogrefe

[39] Brickenkamp R, Lothar Schmidt-Atzert (2010) Detlev Liepmann. d2-R–Aufmerksamkeits-und Konzentrationstest. Hogrefe, Göttingen

[40] Yato Y, Hirose S, Wallon P, Mesmin C, Jobert M (2019) d2-R test for Japanese adolescents: concurrent validity with the attention deficit-hyperactivity disorder rating scale. Pediatr Int 61(1):43–48 30449059 10.1111/ped.13735

[41] Morisky DE, Green LW, Levine DM (1986) Concurrent and predictive validity of a self-reported measure of medication adherence. Med Care. ; 24(1):67–74. Available from: URL: https://pubmed.ncbi.nlm.nih.gov/3945130/10.1097/00005650-198601000-00007 3945130

[42] Shrier I, Steele RJ, Verhagen E, Herbert R, Riddell CA, Kaufman JS (2014) Beyond intention to treat: what is the right question? Clin Trials 11(1):28–37 24096636 10.1177/1740774513504151

[43] Kajiya K, Inaki K, Tanaka M, Haga T, Kataoka H, Touhara K (2001) Molecular bases of odor discrimination: Reconstitution of olfactory receptors that recognize overlapping sets of odorants. J Neurosci. ; 21(16):6018–25. Available from: URL: https://www.ncbi.nlm.nih.gov/pmc/articles/PMC6763140/10.1523/JNEUROSCI.21-16-06018.2001 PMC6763140 11487625

[44] Pires ÍdAT, Steffens ST, Mocelin AG, Shibukawa DE, Leahy L, Saito FL et al (2022) Intensive olfactory training in Post-COVID-19 patients: a Multicenter Randomized Clinical Trial. Am J Rhinol Allergy 36(6):780–787 35866202 10.1177/19458924221113124 PMC9309586

[45] Altundag A, Yilmaz E, Kesimli MC (2022) Modified olfactory training is an effective treatment method for COVID-19 Induced Parosmia. Laryngoscope 132(7):1433–1438 35257391 10.1002/lary.30101 PMC9088368

[46] Altundag A (2023) Parosmia and Phantosmia: Managing Quality Disorders. Curr Otorhinolaryngol Rep. :1–8. Available from: URL: https://link.springer.com/article/10.1007/s40136-023-00441-w 10.1007/s40136-023-00441-w PMC9880375 36721659

[47] Olofsson JK, Ekström I, Larsson M, Nordin S (2021) Olfaction and aging: a review of the current state of research and future directions. Iperception 12(3):20416695211020331 34249327 10.1177/20416695211020331 PMC8239976

[48] Vance DE, Del Bene VA, Kamath V, Frank JS, Billings R, Cho D-Y et al Does olfactory training improve brain function and cognition? A systematic review. Neuropsychol Rev 2023:1–37 10.1007/s11065-022-09573-0 PMC9891899 36725781

[49] Wegener B-A, Croy I, Hähner A, Hummel T (2018) Olfactory training with older people. Int J Geriatr Psychiatry 33(1):212–220 28429377 10.1002/gps.4725

[50] Olofsson JK, Ekström I, Lindström J, Syrjänen E, Stigsdotter-Neely A, Nyberg L et al (2020) Smell-based memory training: evidence of olfactory learning and transfer to the visual domain. Chem Senses 45(7):593–600 32645143 10.1093/chemse/bjaa049 PMC7545250

[51] Olofsson JK, Gottfried JA (2015) The muted sense: neurocognitive limitations of olfactory language. Trends Cogn Sci. ; 19(6):314–21. Available from: URL: https://pubmed.ncbi.nlm.nih.gov/25979848/10.1016/j.tics.2015.04.007 PMC4457599 25979848

[52] Fortin NJ, Agster KL, Eichenbaum HB (2002) Critical role of the hippocampus in memory for sequences of events. Nature neuroscience. ; 5(5):458–62. Available from: URL: https://www.ncbi.nlm.nih.gov/pmc/articles/PMC4053170/10.1038/nn834 PMC4053170 11976705

[53] Haehner A, Chen B, Espin M, Haussmann R, Matthes C, Desser D et al (2022) Training with Odors Impacts Hippocampal Thickness in Patients with Mild Cognitive Impairment. Alzheimers Dis. ; 88(2):743–55. Available from: URL: https://pubmed.ncbi.nlm.nih.gov/35694924/10.3233/JAD-220248 35694924

[54] Negoias S, Pietsch K, Hummel T (2017) Changes in olfactory bulb volume following lateralized olfactory training. Brain Imaging Behav. ; 11(4):998–1005. Available from: URL: https://pubmed.ncbi.nlm.nih.gov/27448159/10.1007/s11682-016-9567-9 27448159

[55] Mahmut MK, Musch M, Han P, Abolmaali N, Hummel T (2020) The effect of olfactory training on olfactory bulb volumes in patients with idiopathic olfactory loss. Rhin. ; 58(4):410–2. Available from: URL: https://pubmed.ncbi.nlm.nih.gov/32533766/10.4193/Rhin20.223 32533766

[56] Kim B-Y, Park J, Kim E, Kim B (2020) Olfactory ensheathing cells mediate neuroplastic mechanisms after olfactory training in mouse model. Am J Rhinol Allergy 34(2):217–229 31680531 10.1177/1945892419885036

[57] Moran DT, Jafek BW, Eller PM, Rowley JC (1992) Ultrastructural histopathology of human olfactory dysfunction. Microsc Res Tech. ; 23(2):103–10. Available from: URL: https://pubmed.ncbi.nlm.nih.gov/1421550/10.1002/jemt.1070230202 1421550

[58] Schepens EJA, de Haas CJM, Postma EM, van Dijk B, Boesveldt S, Stegeman I et al (2024) The effect of smell training on COVID-19 induced smell loss. Rhin 10.4193/Rhin23.191 38446154

[59] Pieniak M, Oleszkiewicz A, Avaro V, Calegari F, Hummel T (2022) Olfactory training - Thirteen years of research reviewed. Neurosci Biobehav Rev. ; 141:104853. Available from: URL: https://www.sciencedirect.com/science/article/pii/S0149763422003426 10.1016/j.neubiorev.2022.104853 36064146

[60] Doty RL, Laing DG (2015) Psychophysical measurement of human olfactory function. In: Doty RL (ed) Handbook of Olfaction and Gustation. Wiley, pp 225–260

[61] Stevens JC, Cain WS, Burke RJ (1988) Variability of olfactory thresholds. Chem Senses 13(4):643–653

