# Supplementary material for: Four odorants for olfactory training are enough: a pilot study
Source: Eur Arch Otorhinolaryngol. 2024 Sep 6;281(12):6445–58. doi: 10.1007/s00405-024-08930-4 (PMC11564259; doi:10.1007/s00405-024-08930-4)
Supplement: Supplementary file 2 — Supplementary Material 2 [file 405_2024_8930_MOESM2_ESM.docx]

(Robert Koch Institut, 2024)(Canas et al., 2023)(Welge-Luessen & Hummel, 2013)(Hummel T, Hummel C, Welge-Luessen A, 2013)(“World Medical Association Declaration of Helsinki. Ethical Principles for Medical Research Involving Human Subjects,” 2001)(Whitcroft et al., 2023)(Drews et al., 2022)(Liu et al., 2020)(Hummel et al., 1997)(Oleszkiewicz et al., 2019)(Gudziol et al., 2006)

**Comparison of patients with OD between no OT, 4-item-OT, and 7-item-OT**

**
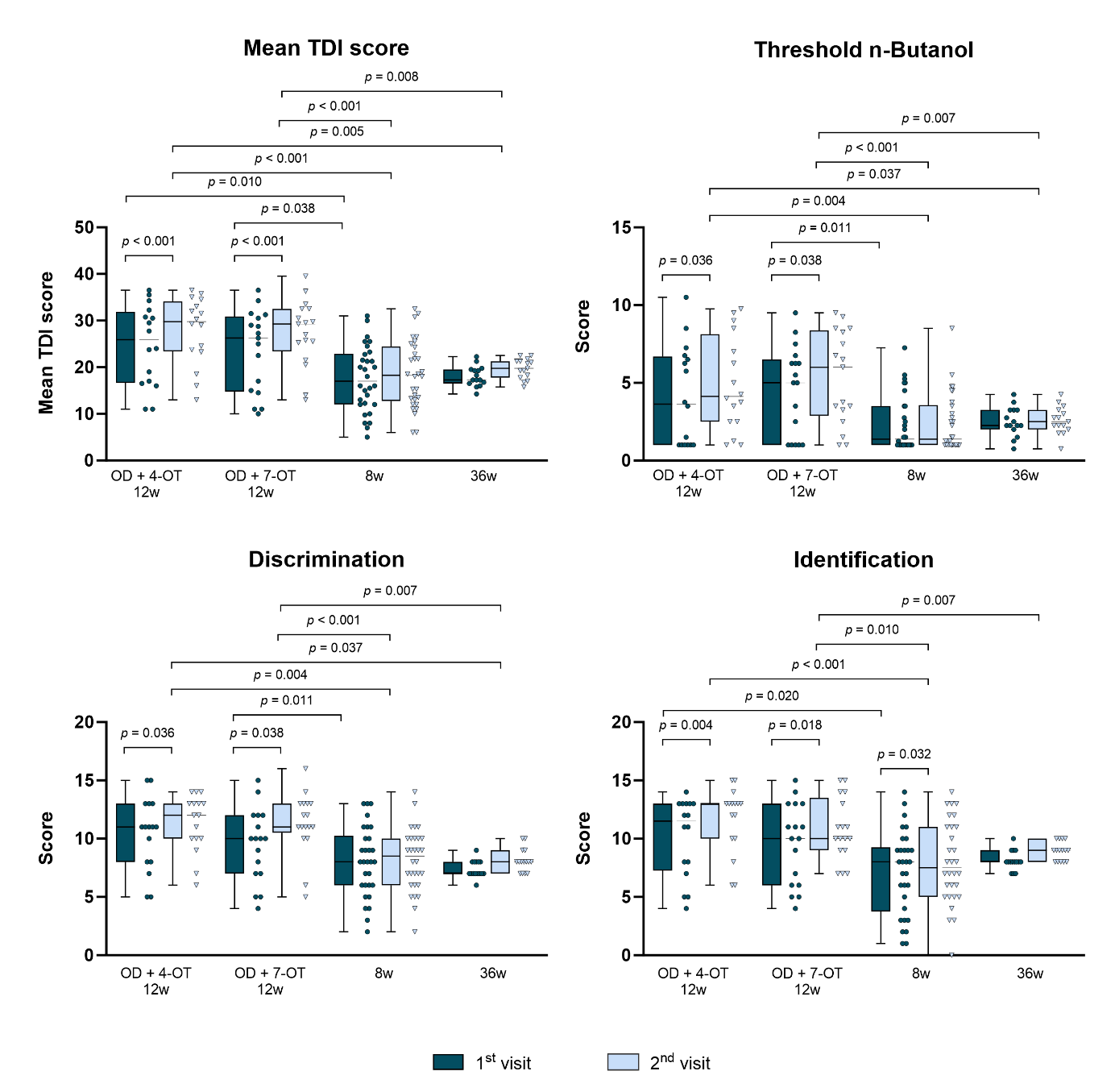
**

**Figure S1:** Comparison of the effect of OT in threshold, discrimination, identification, and combined TDI scores **A**: OT with 4 or 7-item showed an significant increase of the composite TDI score. Patients without OT measured after 8 or 36 weeks showed no improvement. **B–C**: Scores of threshold, discrimination and identification tests. OD + 4-OT *n* = 16, OD + 7-OT *n* = 17, 8w *n* = 30, 36w *n* = 15. Data is represented as median ± SD. Data of the 8 week-group was obtained from Drews et al. (2022). Data of the 36 week-group was retrieved from Liu et al. (2020). Non-significant comparison are not shown. Abbreviations: OD, olfactory dysfunction; 4-OT, 4 item olfactory training; 7-OT, 7 item olfactory training; 8w, patients without OT and a gap of 8 weeks between 1^st^ and 2^nd^ visit; 36w, patients without OT and a gap of 36 weeks between 1^st^ and 2^nd^ visit.

**Differences after and before olfatory training**


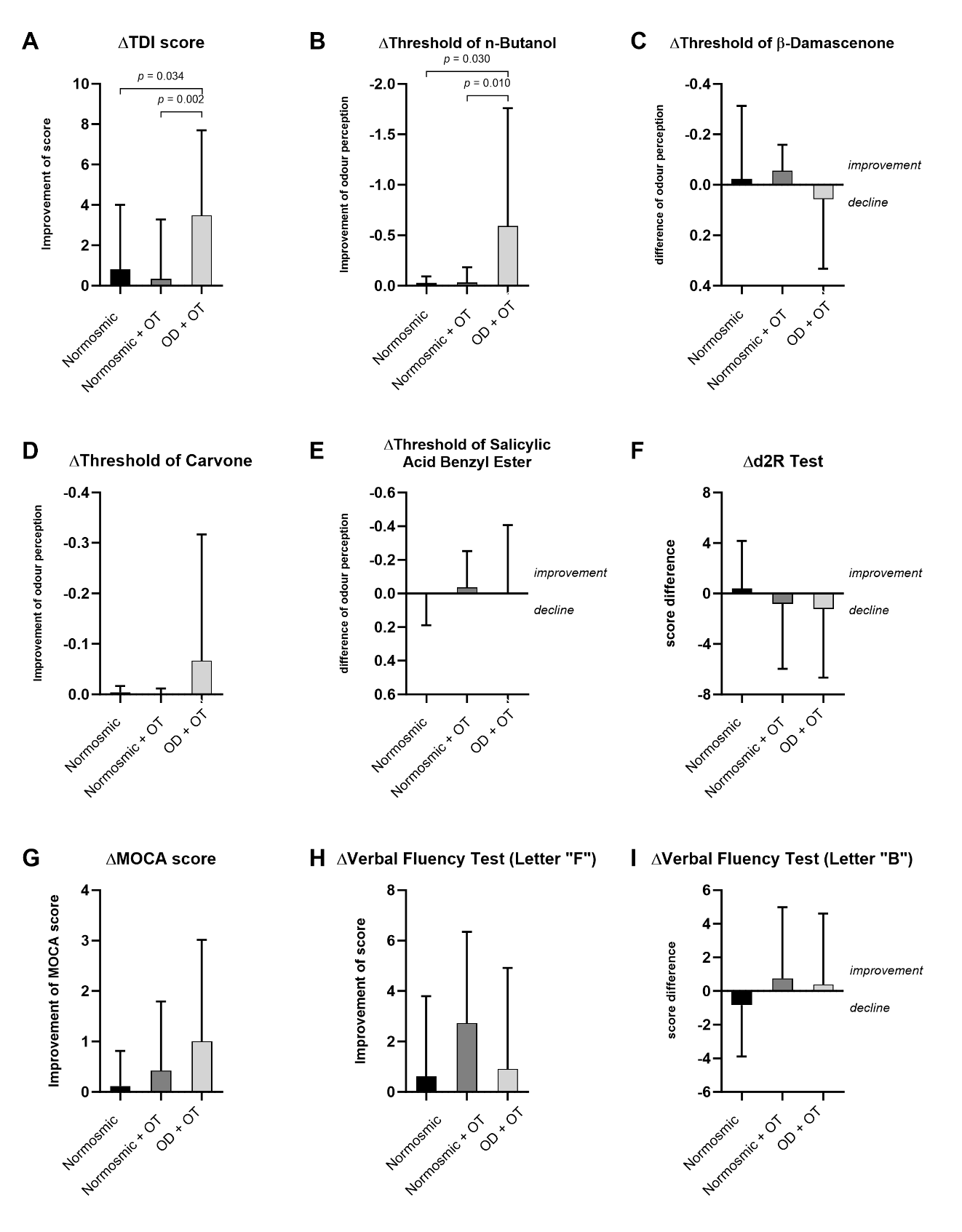
**Figure S2:** Differences after and before OT are shown for **A** TDI score, **B** threshold of n-butanol, **C** β-damascenone, **D** carvone, **E** salicylic acid benzyl ester, **F** MOCA score, **G** d2-R test, **H** verbal fluency test with letter “F”, and **I** verbal fluency test with letter “B”. Data is represented as mean ± SD with. Non-significant comparison are not shown. Abbreviations: OT, olfactory training; OD, olfactory dysfunction.

**Ratings about olfactory ability from patients with qualitative OD**


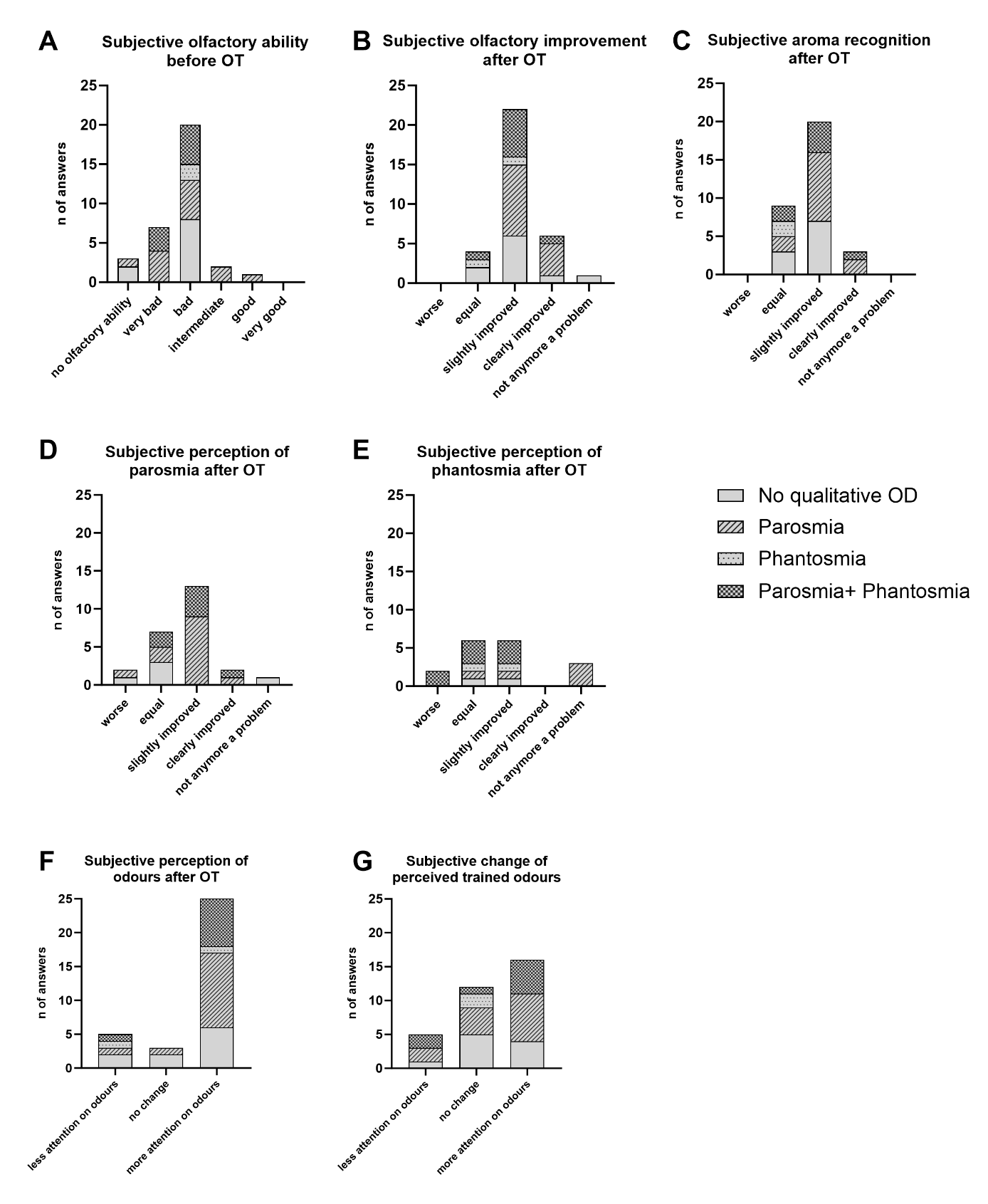


**Figure S3:** Ratings about olfactory ability from patients with qualitative OD. **A**: Subjective olfactory ability was assessed with in a standardized medical interview. After OT, subjective olfactory improvement (**B**), aroma recognition (**C**), perception of parosmia (**D**) and phantosmia (**E**) was assessed in a 6-point Likert-type scale. Additional ratings regarding the perception of odors after OT (**F**) and the trained odors (**G**) was gathered. Data is presented as amount of answer for each question.

**
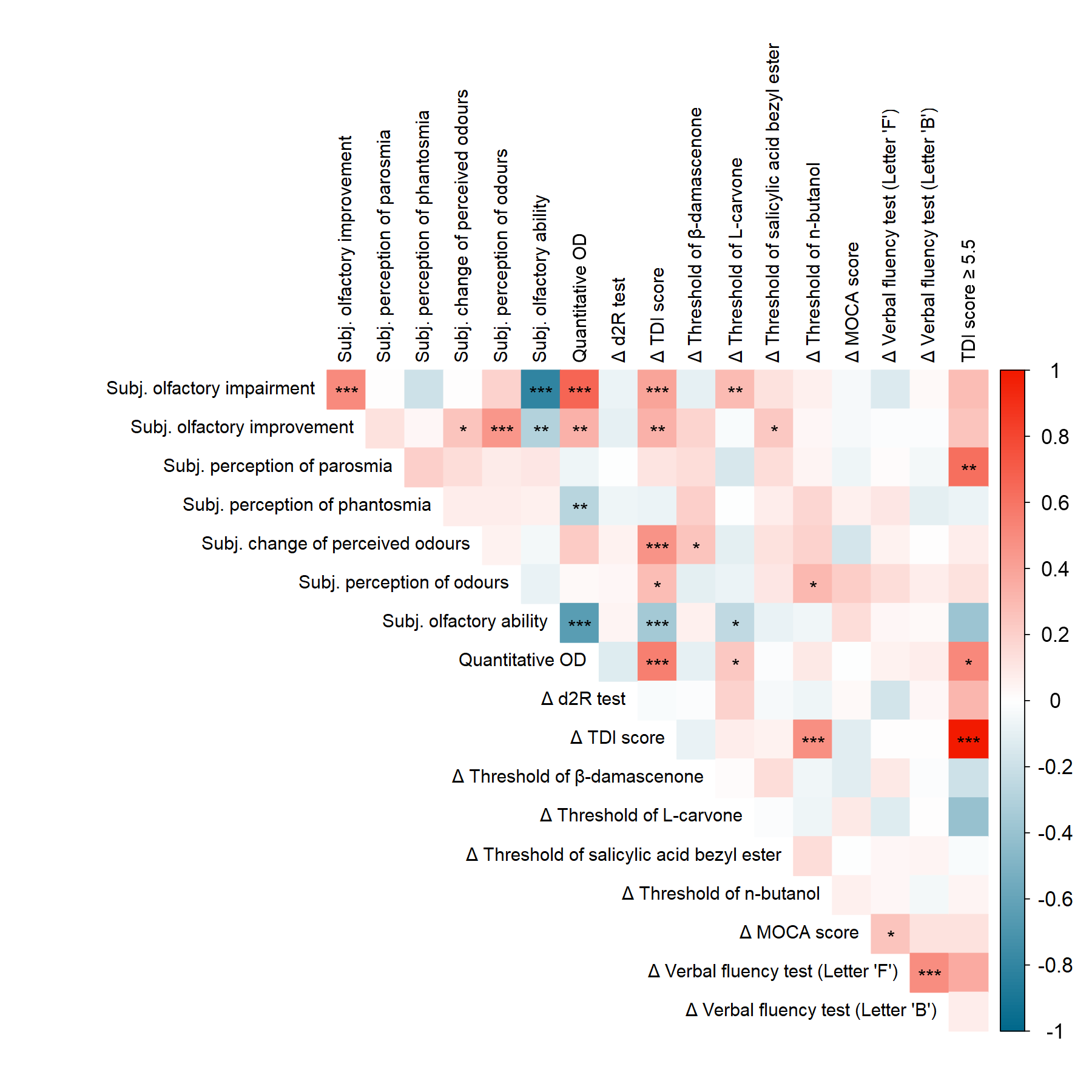
**

**Figure S4:** Pearson’s correlation matrix. Strong positive correlations are indicated in red (1.00-0.70), negative strong correlation in blue (-0.70 – -1.00), or moderate correlations in light colours (above 0.40 or under -0.40; RStudio 2022.07.1+554; https://cran.r-project.org/). The significance level is plotted p-values as *, ** or *** with * p < 0.05; ** p < 0.01; *** p < 0.001. Missing p-values show no significant correlation between two variables. Abbreviations: OT, olfactory training; Δ, difference between after (T1) and before OT (T0); TDI, composite threshold, discrimination and identification score; Subj., subjective; OD, olfactory dysfunction.

References

Brickenkamp, R. (1962). *Test d2: aufmerksamkeits-belastungs-test*. C. J. Hogrefe.

Brickenkamp, R., Lothar Schmidt-Atzert, & Detlev Liepmann. (2010). *d2-R–Aufmerksamkeits-und Konzentrationstest.*

Canas, L. S., Molteni, E., Deng, J., Sudre, C. H., Murray, B., Kerfoot, E., Antonelli, M., Rjoob, K., Capdevila Pujol, J., Polidori, L., May, A., Österdahl, M. F., Whiston, R., Cheetham, N. J., Bowyer, V., Spector, T. D., Hammers, A., Duncan, E. L., Ourselin, S., . . . Modat, M. (2023). Profiling post-COVID-19 condition across different variants of SARS-CoV-2: A prospective longitudinal study in unvaccinated wild-type, unvaccinated alpha-variant, and vaccinated delta-variant populations. *The Lancet Digital Health*, *5*(7), e421-e434. https://doi.org/10.1016/S2589-7500(23)00056-0

Drews, T., Hummel, T [Thomas], Rochlitzer, B., Hauswald, B., & Hähner, A [Antje] (2022). Acupuncture is associated with a positive effect on odour discrimination in patients with postinfectious smell loss-a controlled prospective study. *European Archives of Oto-Rhino-Laryngology*, *279*(3), 1329–1334. https://doi.org/10.1007/s00405-021-06872-9

Gudziol, V., Lötsch, J., Hähner, A [Antje], Zahnert, T., & Hummel, T [Thomas] (2006). Clinical significance of results from olfactory testing. *The Laryngoscope*, *116*(10), 1858–1863. https://doi.org/10.1097/01.mlg.0000234915.51189.cb

Hummel, T [T.], Sekinger, B., Wolf, S. R., Pauli, E., & Kobal, G. (1997). 'sniffin' sticks': Olfactory performance assessed by the combined testing of odor identification, odor discrimination and olfactory threshold. *Chemical Senses*, *22*(1), 39–52. https://doi.org/10.1093/chemse/22.1.39

Hummel T, Hummel C, Welge-Luessen A. (2013). Assessment of Olfaction and Gustation. In A. Welge-Luessen & T. Hummel (Eds.), *Management of Smell and Taste Disorders* (p. 58-75) [Thieme].

Liu, D. T., Pellegrino, R., Sabha, M., Aytug, A., Damm, M., Poletti, S. C., Croy, I., Hähner, A [A.], Oleszkiewicz, A [A.], Mandy, C., & Hummel, T [T.] (2020). Factors associated with relevant olfactory recovery after olfactory training: a retrospective study including 601 participants. *Rhinology Journal*, *0*(0), 0. https://doi.org/10.4193/Rhin20.262

Morisky, D. E., Green, L. W., & Levine, D. M. (1986). Concurrent and predictive validity of a self-reported measure of medication adherence. *Medical Care*, *24*(1), 67–74. https://doi.org/10.1097/00005650-198601000-00007

Nasreddine, Z. S., Phillips, N. A., Bédirian, V., Charbonneau, S., Whitehead, V., Collin, I., Cummings, J. L., & Chertkow, H. (2005). The Montreal Cognitive Assessment, MoCA: A brief screening tool for mild cognitive impairment. *Journal of the American Geriatrics Society*, *53*(4), 695–699. https://doi.org/10.1111/j.1532-5415.2005.53221.x

Oleszkiewicz, A [A.], Schriever, V. A., Croy, I., Hähner, A [A.], & Hummel, T [Thomas] (2019). Updated Sniffin' Sticks normative data based on an extended sample of 9139 subjects. *European Archives of Oto-Rhino-Laryngology*, *276*(3), 719–728. https://doi.org/10.1007/s00405-018-5248-1

Pieniak, M., Oleszkiewicz, A [Anna], Avaro, V., Calegari, F., & Hummel, T [Thomas] (2022). Olfactory training - Thirteen years of research reviewed. *Neuroscience and Biobehavioral Reviews*, *141*, 104853. https://doi.org/10.1016/j.neubiorev.2022.104853

Robert Koch Institut. (2024, January 5). *SARS-CoV-2 Varianten in Deutschland: Daten aus der integrierten genomischen Surveillance von SARS-CoV-2*. https://public.data.rki.de/t/public/views/IGS_Dashboard/DashboardVOC?%3Aembed=y&%3AisGuestRedirectFromVizportal=y

Saatci, O., Altundag, A [Aytug], Duz, O. A., & Hummel, T [Thomas] (2020). Olfactory training ball improves adherence and olfactory outcomes in post-infectious olfactory dysfunction. *European Archives of Oto-Rhino-Laryngology*, *277*(7), 2125–2132. https://doi.org/10.1007/s00405-020-05939-3

Shao, Z., Janse, E., Visser, K., & Meyer, A. S. (2014). What do verbal fluency tasks measure? Predictors of verbal fluency performance in older adults. *Frontiers in Psychology*, *5*, 772. https://doi.org/10.3389/fpsyg.2014.00772

Shrier, I., Steele, R. J., Verhagen, E., Herbert, R., Riddell, C. A., & Kaufman, J. S. (2014). Beyond intention to treat: What is the right question? *Clinical Trials (London, England)*, *11*(1), 28–37. https://doi.org/10.1177/1740774513504151

Thomann, A. E., Berres, M., Goettel, N., Steiner, L. A., & Monsch, A. U. (2020). Enhanced diagnostic accuracy for neurocognitive disorders: A revised cut-off approach for the Montreal Cognitive Assessment. *Alzheimer's Research & Therapy*, *12*(1), 39. https://doi.org/10.1186/s13195-020-00603-8

Welge-Luessen, A [Antje], & Hummel, T [Thomas] (Eds.). (2013). *Management of Smell and Taste Disorders* [Thieme]. https://doi.org/10.1055/b-002-89586

Whitcroft, K. L., Altundag, A [A.], Balungwe, P., Boscolo-Rizzo, P., Douglas, R., Enecilla, M. L. B., Fjaeldstad, A. W., Fornazieri, M. A., Frasnelli, J., Gane, S., Gudziol, H., Gupta, N., Haehner, A., Hernandez, A. K., Holbrook, E. H., Hopkins, C., Hsieh, J. W., Huart, C., Husain, S., . . . Hummel, T [T.] (2023). Position paper on olfactory dysfunction: 2023. *Rhinology Journal.* Advance online publication. https://doi.org/10.4193/Rhin22.483

World Medical Association Declaration of Helsinki. Ethical principles for medical research involving human subjects. (2001). *Bulletin of the World Health Organization*, *79*(4), 373–374.

Yato, Y., Hirose, S., Wallon, P., Mesmin, C., & Jobert, M. (2019). D2-R test for Japanese adolescents: Concurrent validity with the attention deficit-hyperactivity disorder rating scale. *Pediatrics International : Official Journal of the Japan Pediatric Society*, *61*(1), 43–48. https://doi.org/10.1111/ped.13735
